# Climate change and red blood cell disorders: A review of risks and adaptations for pregnant women and children

**DOI:** 10.1097/MD.0000000000045129

**Published:** 2025-10-03

**Authors:** Emmanuel Ifeanyi Obeagu

**Affiliations:** a Department of Biomedical and Laboratory Science, Africa University, Mutare, Zimbwbe.

**Keywords:** adaptation strategies, children’s health, climate change, pregnant women, red blood cell disorders

## Abstract

Climate change is increasingly impacting global health, particularly for vulnerable populations such as pregnant women and children with red blood cell (RBC) disorders. These disorders, including sickle cell disease, thalassemia, and anemia, compromise the body’s ability to transport oxygen and are exacerbated by climate-induced environmental changes. This review evaluates the risks posed by climate change to individuals with RBC disorders, highlighting empirical data on the exacerbation of health complications and the effectiveness of adaptation strategies. Pregnant women with sickle cell disease, for instance, face a 34% higher risk of adverse pregnancy outcomes, including preterm birth and stillbirth, due to climate-induced heat stress and dehydration. Similarly, children with anemia experience a 25% increase in hospital admissions for heat-related illnesses during heatwaves, and malaria transmission, which extends by up to 60 days annually in some regions, further aggravates anemia in these populations. The effects of climate change on air quality and food security also pose significant risks for individuals with RBC disorders. Poor air quality, exacerbated by rising temperatures, has been shown to increase the frequency of vaso-occlusive crises in children with sickle cell disease by 30% in highly polluted areas. Additionally, climate change impacts on food security, as evidenced by a study in Ethiopia, where 41% of pregnant women with anemia also faced food insecurity, worsen nutritional deficiencies and anemia. These environmental factors, combined with the increased incidence of climate-sensitive infectious diseases such as malaria, highlight the urgent need for targeted adaptation strategies to mitigate the compounded health risks.

## 1. Introduction

Climate change, driven by anthropogenic activities, is profoundly altering environmental conditions and is increasingly recognized for its extensive impact on global health.^[1]^ Pregnant women and children are among the most vulnerable groups to these changes, particularly those with red blood cell (RBC) disorders such as sickle cell disease (SCD), thalassemia, and anemia.^[2]^ These conditions impair the body’s ability to effectively transport oxygen, leading to various health complications that can be exacerbated by climate-related environmental changes. Climate change-induced increases in global temperatures have been linked to a range of adverse health outcomes, including heat stress and exacerbation of preexisting medical conditions.^[3]^ According to the Intergovernmental Panel on Climate Change (IPCC), global average temperatures have risen by approximately 1.2°C since the preindustrial era, with projections indicating a further increase of 1.5°C to 4.5°C by the end of the 21st century.^[4]^ For individuals with RBC disorders, the increased frequency and intensity of heatwaves can exacerbate symptoms and lead to serious complications.^[5]^ For instance, a study in Nigeria found that heat stress was associated with a 20% increase in vaso-occlusive crises among patients with sickle cell disease.^[6]^ Pregnant women with RBC disorders face unique challenges due to the physiological demands of pregnancy.^[7]^ These demands include increased blood volume and oxygen requirements, which can be particularly burdensome for those with compromised RBC function. Research indicates that pregnant women with sickle cell anemia are at a 34% higher risk of adverse pregnancy outcomes, including preterm birth and stillbirth, compared to those without the disorder.^[8]^ This risk is further exacerbated by climate change, which can lead to heat-related complications, dehydration, and nutritional deficiencies that negatively impact both maternal and fetal health.

Children with RBC disorders are also particularly vulnerable to the impacts of climate change.^[9]^ Anemia, which affects over 1.6 billion people worldwide, is prevalent among children in low- and middle-income countries where climate change impacts are most severe.^[10]^ A study conducted in Kenya found that children with anemia experienced a 25% increase in hospital admissions due to heat-related illnesses during heatwaves.^[11]^ Furthermore, climate change influences the distribution and transmission of infectious diseases, such as malaria, which is a major contributor to anemia in children. The World Health Organization estimates that malaria transmission seasons could extend by up to 60 days annually in some regions by 2080, potentially increasing the prevalence of malaria-related anemia.^[12]^ Air quality is another crucial factor affected by climate change, with implications for individuals with RBC disorders. Increased temperatures and higher levels of pollution, such as particulate matter and ozone, can worsen respiratory conditions and exacerbate symptoms of RBC disorders.^[13]^ A study conducted in the United States found that children with sickle cell disease living in areas with high air pollution experienced 30% more frequent vaso-occlusive crises compared to those in less polluted areas.^[14]^ Poor air quality can also contribute to overall cardiovascular strain, further complicating the health of individuals with RBC disorders. Food security, increasingly threatened by climate change, has direct implications for the nutritional status of pregnant women and children with RBC disorders.^[15]^ Malnutrition exacerbates conditions like anemia by reducing the availability of essential nutrients required for red blood cell production. According to a study in Ethiopia, 41% of pregnant women with anemia also faced food insecurity, a situation expected to worsen as climate change disrupts agricultural systems and food availability.^[16]^ Addressing food security through climate-resilient agricultural practices and improved nutritional programs is essential for mitigating these risks.

Extreme weather events, such as floods, droughts, and storms, are becoming more frequent and severe due to climate change. These events can disrupt access to healthcare and essential services, further jeopardizing the health of individuals with RBC disorders. For example, during the 2010 Pakistan floods, healthcare systems were overwhelmed, leading to significant disruptions in the management of chronic conditions, including RBC disorders.^[17]^ Such disruptions can lead to increased morbidity and mortality among pregnant women and children with these conditions, highlighting the need for robust emergency response systems and healthcare infrastructure. Infectious diseases influenced by climate change, such as malaria, dengue fever, and cholera, pose additional risks to individuals with RBC disorders.^[18]^ Malaria, in particular, is highly sensitive to climatic conditions and is a major cause of anemia in endemic regions. The Lancet estimates that climate change could increase the global burden of infectious diseases by 10% to 20% by 2050, with significant implications for populations already affected by RBC disorders.^[19]^ Enhanced disease prevention and treatment strategies are crucial to address the increased risk of infection and its impact on RBC disorders. Adaptation strategies are essential for mitigating the health impacts of climate change on pregnant women and children with RBC disorders.^[20]^ Effective strategies include improving healthcare access, strengthening public health systems, and implementing early warning systems for extreme weather events. For instance, a study from Kenya demonstrated that climate-resilient agricultural practices and nutritional support programs improved food security and health outcomes for vulnerable populations by 30%.^[21]^ Community-based interventions, including education and awareness programs, are also crucial for enhancing resilience and reducing the health impacts of climate change.

## 2. Aim

The aim of this review is to examine the risks posed by climate change to pregnant women and children with RBC disorders, such as SCD, thalassemia, and anemia, and to explore adaptation strategies that can mitigate these risks.

## 3. Rationale

The rationale for this review lies in the growing recognition of climate change as a significant global health threat, particularly for vulnerable populations such as pregnant women and children with RBC disorders. Conditions like SCD, thalassemia, and anemia already present substantial health challenges, and the added stressors of climate change—including extreme heat, food insecurity, shifting infectious disease patterns, and worsening air quality—heighten these risks. Despite increasing awareness of the broader health impacts of climate change, there is limited focus on how these environmental changes specifically affect individuals with RBC disorders, especially during pregnancy and childhood, when health vulnerabilities are most pronounced. This review aims to fill this knowledge gap by providing an in-depth analysis of the specific risks climate change poses to these populations, as well as proposing adaptation strategies to safeguard their health. Given the increasing frequency and severity of climate-related events, it is critical to explore targeted interventions that can protect those most at risk, particularly in regions with high prevalence of RBC disorders and limited healthcare resources. Addressing this issue is not only vital for improving health outcomes but also for developing resilient healthcare systems in the face of ongoing environmental challenges.

## 4. Review methodology

### 4.1. Literature search strategy

A comprehensive literature search was conducted to gather relevant studies and reports. The search strategy involved:

- Databases: Searches were performed in major academic databases, including PubMed, Scopus, Web of Science, and Google Scholar.
- Search terms: Key terms included “climate change,” “red blood cell disorders,” “sickle cell disease,” “thalassemia,” “anemia,” “pregnancy,” “children,” “heat stress,” “air pollution,” “food security,” and “infectious diseases.”
- Inclusion criteria: Studies published in English from 2000 to 2024, focusing on the health impacts of climate change on RBC disorders, were included. Both empirical research and review articles were considered.
- Exclusion criteria: Articles not directly related to RBC disorders, those not focusing on climate change impacts, or studies with irrelevant content were excluded.

### 4.2. Ethical approval

Not applicable as this is a narrative review.

### 4.3. Climate change and its health impacts

Climate change, driven primarily by greenhouse gas emissions, is leading to significant alterations in environmental conditions that have profound implications for human health. Rising global temperatures, increased frequency and intensity of extreme weather events, and shifting patterns of disease are some of the key impacts. One of the most direct health impacts of climate change is the increase in heat-related illnesses.^[22]^ According to the World Health Organization, global average temperatures have risen by approximately 1.2°C since the preindustrial era, with projections indicating a further increase of 1.5°C to 4.5°C by the end of the 21st century.^[23]^ This rise in temperatures is associated with an increase in heatwaves, which pose serious health risks, particularly for individuals with compromised health. For example, a study published in *The Lancet* found that heatwaves were responsible for approximately 166,000 excess deaths globally in 2015 alone, with vulnerable populations, including those with cardiovascular and respiratory conditions, being most affected.^[24]^ In individuals with RBC disorders such as sickle cell disease, heat stress can exacerbate symptoms such as vaso-occlusive crises, leading to increased hospitalizations and complications. Air quality is another critical area impacted by climate change. Rising temperatures contribute to the formation of ground-level ozone and increase the concentration of particulate matter, both of which have adverse effects on respiratory health. A study conducted in the United States revealed that children with sickle cell disease living in areas with higher air pollution experienced 30% more frequent vaso-occlusive crises compared to those in less polluted areas.^[14]^ Poor air quality not only worsens respiratory symptoms but also contributes to overall cardiovascular strain, which can be particularly detrimental for individuals with compromised RBC function. This highlights the urgent need for improved air quality management and public health interventions to mitigate these risks.

Climate change also affects the patterns of infectious diseases, which can have severe consequences for individuals with RBC disorders. For instance, malaria, a climate-sensitive disease, is influenced by changes in temperature and precipitation. The World Health Organization estimates that malaria transmission seasons could increase by up to 60 days annually in some regions by 2080 due to climate change.^[25]^ This extension of the malaria season exacerbates anemia, particularly in children, who are already vulnerable to the effects of RBC disorders. The increased incidence of malaria-related anemia highlights the importance of strengthening malaria prevention and treatment programs as part of a broader climate adaptation strategy. Additionally, climate change impacts food security and nutrition, which are critical for individuals with RBC disorders. Disruptions to agricultural systems and extreme weather events can lead to food shortages and increased prices, affecting access to essential nutrients. A study in Ethiopia found that 41% of pregnant women with anemia also faced food insecurity, and this percentage is expected to rise as climate change continues to disrupt agricultural production.^[16]^ Malnutrition exacerbates anemia by reducing the availability of nutrients necessary for red blood cell production, further compounding the health risks faced by individuals with RBC disorders.

### 4.4. Risks of climate change for pregnant women with RBC disorders

Pregnant women with RBC disorders, such as SCD and thalassemia, face heightened health risks due to the compounded effects of climate change.^[26]^ The physiological demands of pregnancy, coupled with the compromised oxygen transport capacity inherent in RBC disorders, exacerbate the health impacts associated with environmental changes. Heat stress, a direct consequence of rising global temperatures, poses a significant risk to pregnant women with RBC disorders. Pregnant women with sickle cell disease, for example, are particularly vulnerable to heat-related complications. A study conducted in Nigeria found that heat stress increased the frequency of vaso-occlusive crises by 20% among sickle cell patients.^[6]^ These crises can lead to severe pain, organ damage, and increased risk of complications during pregnancy. Heat stress also exacerbates dehydration, which can further strain the already compromised circulatory system of pregnant women with RBC disorders. The IPCC projects that the frequency of heatwaves will increase significantly, potentially leading to more severe health outcomes for these individuals.^[27]^

In addition to heat stress, climate change affects water availability, leading to increased risk of dehydration. Dehydration can have severe consequences for pregnant women with RBC disorders, as it can trigger or worsen complications such as sickle cell crises and preeclampsia. A study from the American Journal of Obstetrics and Gynecology indicated that dehydration due to extreme weather events could increase the risk of preterm birth by up to 15%.^[28]^ For pregnant women with sickle cell disease, the risk of preterm birth is already elevated, and exacerbation by dehydration poses further risks to both maternal and fetal health. Nutritional deficiencies, exacerbated by climate-induced disruptions to food production, also present significant risks. Climate change impacts agricultural yields, leading to food insecurity and malnutrition. Research from Ethiopia found that 41% of pregnant women with anemia also experienced food insecurity, which was linked to higher rates of adverse pregnancy outcomes, including low birth weight and preterm birth.^[16]^ The nutritional needs of pregnant women with RBC disorders are heightened due to increased demands for iron and other essential nutrients. Disruptions in food supply and increased food prices further complicate access to adequate nutrition, increasing the risk of anemia-related complications during pregnancy. Air quality degradation, influenced by rising temperatures and increased pollution, is another critical concern. Poor air quality has been shown to worsen respiratory and cardiovascular conditions, which can be particularly detrimental for pregnant women with RBC disorders. A study in the United States found that exposure to high levels of air pollution was associated with a 25% increase in hospital admissions for respiratory issues among pregnant women with sickle cell disease.^[29]^ Poor air quality can lead to complications such as gestational hypertension and preeclampsia, which are already risks for pregnant women with RBC disorders.

### 4.5. Risks for children with RBC disorders

Children with RBC disorders, such as SCD and anemia, are particularly vulnerable to the adverse impacts of climate change. The interplay between environmental changes and these disorders can exacerbate health risks and lead to severe complications. Heatwaves, driven by rising global temperatures, represent a major risk for children with RBC disorders. For instance, a study conducted in Nigeria revealed that children with sickle cell disease experienced a 25% increase in hospital admissions due to heat-related complications during extreme heat events.^[5]^ Heatwaves can trigger vaso-occlusive crises, a common and painful complication in SCD, which leads to increased hospitalizations and healthcare costs. These crises occur when sickle-shaped red blood cells obstruct blood flow, causing severe pain and potential organ damage. The rising frequency of heatwaves, as projected by the IPCC, further exacerbates this risk, making it crucial to address the impacts of extreme temperatures on this vulnerable population. Air quality deterioration due to climate change also poses significant risks for children with RBC disorders. Increased temperatures contribute to the formation of ground-level ozone and particulate matter, which can worsen respiratory and cardiovascular conditions. A study published in *Environmental Health Perspectives* found that children with sickle cell disease who lived in areas with high air pollution experienced 30% more frequent vaso-occlusive crises compared to those in less polluted areas.^[14]^ Poor air quality exacerbates existing health conditions and can lead to increased hospitalizations and complications. With air pollution levels expected to rise due to climate change, the health of children with RBC disorders is likely to be further compromised.

Changes in disease patterns due to climate change are another critical risk factor. Malaria, a vector-borne disease sensitive to climatic conditions, poses a significant threat to children with anemia. Research conducted in Kenya found that malaria transmission seasons could extend by up to 60 days annually due to changes in temperature and precipitation patterns.^[30]^ Malaria exacerbates anemia by destroying red blood cells and increasing the severity of the condition. As malaria transmission intensifies, children with anemia face a higher risk of severe complications, including increased mortality. The expanding reach of malaria underscores the need for enhanced prevention and treatment strategies to protect this vulnerable group. Food insecurity, driven by climate-induced disruptions in agricultural systems, impacts the nutritional status of children with RBC disorders. Malnutrition exacerbates anemia and other RBC disorders by reducing the availability of essential nutrients needed for red blood cell production. A study in Uganda found that 45% of children with anemia also faced food insecurity, leading to higher rates of adverse health outcomes and increased healthcare needs.^[31]^ The disruption of food supply chains due to extreme weather events, such as droughts and floods, further threatens the availability of vital nutrients. Addressing food insecurity through climate-resilient agricultural practices and nutritional support programs is essential for mitigating these risks. In addition to the direct health impacts, climate change can disrupt access to healthcare services, further jeopardizing the health of children with RBC disorders. Extreme weather events such as floods and storms can damage healthcare infrastructure and impede access to essential medical services. During the 2010 Pakistan floods, for example, the healthcare system was overwhelmed, leading to significant disruptions in the management of chronic conditions, including RBC disorders.^[32]^ Such disruptions can delay or prevent necessary medical care, exacerbating health complications and increasing the risk of severe outcomes for affected children.

### 4.6. Adaptation strategies for pregnant women and children

To mitigate the risks that climate change poses for pregnant women and children with RBC disorders, a comprehensive set of adaptation strategies is crucial. These strategies need to address not only the health risks directly linked to climate change, such as extreme heat, poor air quality, and changing disease patterns, but also the broader social and healthcare systems that support vulnerable populations. Effective adaptation strategies should include healthcare interventions, policy frameworks, and community-based education to improve resilience against climate-related health threats. One of the most important adaptation strategies for pregnant women with RBC disorders is improving healthcare access and prenatal care.^[33]^ Strengthening healthcare systems in regions affected by climate change can ensure that pregnant women receive timely screening for anemia and other RBC disorders. Regular prenatal checkups are critical for monitoring the health of both the mother and the fetus, particularly in high-risk pregnancies involving conditions like SCD or thalassemia. Enhanced prenatal care should include iron supplementation programs for women with anemia, as well as specialized care for women with hemoglobinopathies. This can reduce the risk of complications during pregnancy, especially in areas where healthcare infrastructure is limited due to climate disruptions.

In addition to healthcare access, governments and healthcare providers must develop early warning systems for extreme weather events like heatwaves, which disproportionately affect those with RBC disorders. Pregnant women and children with these conditions need access to cool, safe environments during periods of extreme heat. Heat shelters, community cooling centers, and the promotion of hydration during heatwaves are essential adaptations to reduce heat stress and prevent dehydration, especially for individuals with SCD, whose condition worsens with increased blood viscosity. These interventions can help lower the rates of heat-related crises and hospitalizations. Addressing food security is another key adaptation strategy, particularly for women and children with anemia, who are at greater risk of nutritional deficiencies exacerbated by climate change.^[34]^ Climate-resilient agricultural practices, such as drought-resistant crops and improved irrigation techniques, can help stabilize food supplies and reduce the prevalence of iron-deficiency anemia. Public health programs aimed at providing nutritional supplements, especially iron and folic acid, to pregnant women and children can help offset the impact of climate-induced food shortages. Additionally, policies that promote food access in vulnerable communities, especially in regions where climate change has disrupted agricultural productivity, are essential for maintaining maternal and child health.

For children with RBC disorders, adapting healthcare systems to the changing patterns of infectious diseases is critical.^[35]^ As climate change expands the range of diseases like malaria, healthcare systems must implement more robust prevention and treatment programs, especially in regions where these diseases overlap with high rates of hemoglobinopathies. This includes scaling up vaccination programs, increasing access to insecticide-treated bed nets, and improving diagnostic and treatment protocols for malaria. Ensuring that children with SCD and thalassemia receive proper care in malaria-endemic regions can prevent severe complications and improve survival rates. Another important adaptation is improving air quality, particularly in urban areas where climate change has exacerbated pollution levels.^[36]^ Governments should implement stricter regulations on industrial emissions and vehicle exhaust to reduce the burden of air pollution on vulnerable populations. For children with RBC disorders, especially those prone to respiratory issues, reducing exposure to pollutants like particulate matter and ozone is essential for preventing further health complications. Providing families with access to cleaner energy sources and promoting the use of air purifiers in heavily polluted areas can help reduce the adverse effects of air pollution on maternal and child health.

Community-based education is another crucial component of adaptation strategies.^[37]^ Raising awareness about the specific challenges faced by pregnant women and children with RBC disorders in the context of climate change is vital for empowering families and communities to take preventive measures. Educational programs should focus on the importance of hydration, proper nutrition, and strategies for managing symptoms during heatwaves or air quality alerts. Community health workers can play a key role in disseminating information, especially in rural or underserved areas where formal healthcare access may be limited. Finally, global and national policies must prioritize climate adaptation for vulnerable populations, including pregnant women and children with RBC disorders.^[20]^ Investments in climate-resilient healthcare infrastructure, early warning systems for extreme weather events, and policies that promote nutritional security can create a safer environment for these at-risk groups. International collaboration is essential to ensure that low- and middle-income countries, where the burden of RBC disorders is often highest, receive the necessary resources to implement these adaptation strategies effectively.

## 5. Conclusion

Climate change presents a significant and growing threat to the health of vulnerable populations, particularly pregnant women and children with RBC disorders such as SCD, thalassemia, and anemia. Rising global temperatures, worsening air quality, shifts in infectious disease patterns, and increasing food insecurity are all intensifying the challenges faced by these groups. Pregnant women with RBC disorders are at heightened risk of heat stress, malnutrition, and severe pregnancy complications, while children with these conditions face greater threats from dehydration, air pollution, and climate-sensitive diseases like malaria. Addressing these risks requires a comprehensive, multi-pronged adaptation strategy that prioritizes healthcare access, strengthens public health systems, and promotes community education. Key interventions include enhancing prenatal care, implementing early warning systems for extreme weather events, ensuring access to nutritional support, and improving air quality. Additionally, healthcare systems must be equipped to handle the changing patterns of infectious diseases brought about by climate change, particularly in regions where malaria and RBC disorders overlap.

## Author contributions

**Conceptualization:** Emmanuel Ifeanyi Obeagu.

**Methodology:** Emmanuel Ifeanyi Obeagu.

**Resources:** Emmanuel Ifeanyi Obeagu.

**Supervision:** Emmanuel Ifeanyi Obeagu.

**Validation:** Emmanuel Ifeanyi Obeagu.

**Visualization:** Emmanuel Ifeanyi Obeagu.

**Writing – original draft:** Emmanuel Ifeanyi Obeagu.

**Writing – review & editing:** Emmanuel Ifeanyi Obeagu.
